# Retraction: Bioheat transfer model of transcutaneous spinal cord stimulation-induced temperature changes

**DOI:** 10.7717/peerj.4921/retraction

**Published:** 2018-08-10

**Authors:** PeerJ Editorial Office

Retraction Notice: Chen L, Ke A, Zhang P, Gao Z, Zou X, He J. (2018) Bioheat transfer model of transcutaneous spinal cord stimulation-induced temperature changes. PeerJ 6:e4921 https://doi.org/10.7717/peerj.4921

The authors wish to Retract this article. Upon publication it was realized that certain research collaborators had not been appropriately notified of the publication and therefore they had not been able to provide input into the article, nor to approve the final publication. All authors on this article agree to this Retraction.

